# Research on high proportion of clean energy grid-connected oscillation risk prediction technology based on CNN and trend feature analysis

**DOI:** 10.1038/s41598-023-49634-9

**Published:** 2023-12-13

**Authors:** Wang Pu, Xie Yingnan, Zhao Chongjuan, Shi Hong, Fan Yingwei, Lu Yunfeng, Ding Han, Jin Ye, Yan xueying, Hu yuying

**Affiliations:** 1 State Grid Huzhou Electric Power Supply Company, Huzhou, China; 2 Huzhou Electric Power Design Institute Co., Huzhou, China

**Keywords:** Renewable energy, Electrical and electronic engineering, Energy infrastructure

## Abstract

Oscillations, commonly known as a universal, propagative, and intricate event in the new power system, often give rise to generator tripping and load shedding, not only adversely affecting the power flow limit and the power angle stability but also posing threats to the lines of defense for stability and protection. Traditionally, emphasis has been laid on post-fault oscillation management, an emergency measure to deal with the impact and damage that have already affected the power grid. As such, this paper focuses on an oscillation prediction technique to detect oscillation energy early and intervene proactively to prevent further faults. This technique effectively lessens the damage caused by impacts and disconnects to the power grid. Firstly, this paper proposes the concept of disturbance power density and establishes the correlation between disturbance energy and the time domain, thereby exploring a method for evaluating the pattern of electrical quantities before power system oscillation. Secondly, it speeds up the time it takes to detect faults by catching nuances of voltage-current phase angle and impedance. Lastly, it puts forward a technique to cope with the intricacy and variety of power grid equipment using the convolutional neural network (CNN). This technique incorporates an integrated attention mechanism within a one-dimensional CNN model to capture the implicit mapping between voltage, active power, and reactive power at any time in the power system. This enables the model to self-learn multi-device characteristics and enhances the possibility of using theory in practical ways. Moreover, practical case studies also show that the prediction technique proposed in this paper can effectively issue warnings eight minutes before the occurrence of oscillation.

## Introduction

### motivation and incitement

**Figure 1:**
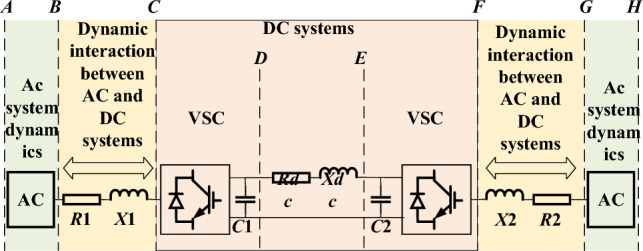
Schematic diagram of DC system.

The oscillation problems of traditional power systems mainly include negative damping oscillations caused by heavy load lines, modern fast excitation and high cap multiple excitation systems^1^, and sub-synchronous resonance/oscillation (SSR/SSO) caused by induction generator effect, shafting torsional vibration and transient torque amplification^2^. In modern "double-high" power systems, a large number of renewable energy units with low inertia, weak stability, weak immunity and output randomness interact with existing power generation equipment, transmission network, power load and themselves, bringing about a variety of new oscillation problems^3^. This type of oscillation is dominated by converter control, with complex excitation mechanism and wide frequency range (order of 10^–1^ ~ 10^3^ Hz)^4^. Therefore, in the modern power system, the traditional low-frequency oscillation, SSR/SSO and the new type of oscillation led by the converter exist at the same time, threatening the safe and stable operation of the power system. As shown in Fig. 1, it is a typical DC system for wind power new energy^3–5^.

Take Guyuan Wind Farm in China as an example. Guyuan Wind Farm is located in North China. The main model of the wind farm is 1.5 MW Double fed induction generator (DFIG), supplemented by a small number of PMSG and constant speed induction generators^6^. The wind farm is distributed in an area of thousands of square kilometers. It is collected to Guyuan transformer station through radiation-type grid, and connects to western Inner Mongolia Power Grid and North China Power grid through two series complement transmission lines with 40% (Hanhai-Guyuan double cycle) and 45% (Guyuan Taiping double cycle) respectively. From the end of 2012 to the beginning of 2014, records show that 58 times of synchronous resonance occurred in the power grid of Guyuan area, and in some serious cases, a large area of fans even went off-grid. Taking the sub-synchronous resonance event on December 25, 2012 as an example, the accident lasted for 8 min and could be divided into three stages: In the first stage, the initial stage of the accident, the output current of the fan diverges rapidly, the amplitude exceeds 30%, and the oscillation frequency is 7.9 Hz In the second stage: fan protection trip, part of the fan is off the network, the system continues to oscillate, the oscillation frequency drops to 6.2 Hz; In the third stage, continue to remove part of the fan and the oscillation is finally suppressed^7^.

### Literature review

In the current project, the oscillation evaluation is mainly based on the method of disturbance power statistics^6,8^. The core of this method is to screen out the values with high disturbance intensity and count the number of times by setting a threshold. After reaching a certain number of statistics, it identifies the instability of the junction point. However, this method has two shortcomings: 1: The power output of new energy itself is volatile, and due to the popularity of high-power charging piles, the load fluctuation characteristics are more significant, so it is difficult to set a reasonable threshold, and the power mutation is obvious. 2: The grid oscillation is a systematic process, which is jointly determined by the impedance phase angle and mutation amount^4^. If only the disturbance amount is concerned, the lead of risk assessment will be greatly shortened. There are also scholars who analyze the grid structure at the theoretical level and predict the stability of the grid from the perspective of RLC model through data modeling. However, in the actual power grid operation process, especially the distribution network structure, its topology and load structure can be flexibly changed, so the RLC model cannot adapt to the real time power grid^9^. In the aspect of neural network technology, the risk assessment of oscillation and the intensity of junction points are also important research fields. In the power grid with regular signal and slow change, the neural network can effectively realize the oscillation risk assessment through online data learning. However, for the junction points of new energy grid with obvious fluctuations, the training data is unable to achieve a stable convergence coefficient^10^.

In this context, artificial intelligence provides a new way to solve the broadband oscillation problem of power system because of its low dependence on the system model, strong learning ability of nonlinear complex relationship between large amounts of data and rapid adaptability to random time-varying environments. Throughout the history of the development of artificial intelligence, it can be found that it has continuously enriched its own methods in the process of development, and gradually formed a system suitable for solving the problem of broadband oscillation. As an important part of data science, artificial intelligence can efficiently extract useful information from massive data and reveal the inherent laws of complex systems through data analysis and mining, thus avoiding the problem of accurate modeling of actual high-dimensional power systems^11^. With the proposed deep learning algorithm of neural networks in 2006, using the powerful learning ability and expression ability of multi-layer networks and a large number of neurons, artificial intelligence methods can realize the approximation of any complex nonlinear mapping relationship^12^, providing a new analysis method for wide-band oscillation problems with significant nonlinearity. Then, in 2013, DeepMind published a paper on using reinforcement learning to play Atari games^13^, and reinforcement learning began a decade of rapid development. The emergence of reinforcement learning has greatly improved the adaptability of artificial intelligence to random time-varying "double high" power systems. Therefore, the artificial intelligence developed so far has the ability to deal with broadband oscillations in methods and technologies.

In literature^14^, Graph Convolutional Network (GCN) algorithm is combined with Long Short-Term Memory (LSTM) algorithm to make full use of spatial and temporal information, but the time of fault removal is unknown in the actual process. Although training only for the features of a single fault removal moment can achieve good results and fast output, it cannot be applied to the actual system. And the trained GCN model is difficult to realize the migration from the source domain to the target domain through migration learning when facing the new system. In literature^15^, the limiting resection time of transient stability is obtained and the stability margin and prediction results are obtained by using Elastic Net. However, the input only considers the steady-state characteristics, which is difficult to accurately characterize the transient boundary. Moreover, the characterization ability of linear regression algorithm is limited in the face of high-dimensional nonlinear characteristics. There are many and relatively mature studies on transient power Angle stability assessment. In literature^16^, rotor locus cluster features are extracted and deep confidence Data Base Network (DBN) is introduced to predict transient power Angle stability and the stability margin is described by rotor Angle disturbance degree. In literature^17^, feature extraction is carried out by Stacked Denoising Auto Encoder (SDAE) and confidence degree is introduced. The mapping between transient power Angle features and stability results is constructed by ensemble learning, but it is difficult to accurately quantify the stability or instability margin relying only on the disturbance degree and confidence of rotor Angle locus cluster. To sum up, there are some problems in artificial intelligence-based assessment of transient power Angle and voltage security and stability, such as long observation time window required for prediction, poor applicability of evaluation indexes of transient safety margin, and inability to distinguish dominant instability modes. In addition, most studies separate transient voltage stability from transient power Angle stability, but ignore the interlacing effects of power Angle and voltage problems in practice. Therefore, it is difficult to clearly describe the overall security of the system.

### Contribution and paper organization

Firstly, this paper constructs a model to examine the stability of power-electronized power systems, explores the mechanisms and characteristics of harmonic disturbances in these systems, and analyzes the challenges associated with low rotational inertia and considerable output fluctuations when integrating renewable energy into the grid. Secondly, it introduces the concept of disturbance power density and defines the correlation between disturbance energy and the time domain, thereby exploring a method for evaluating the pattern of electrical quantities before power system oscillation. By monitoring variations in the voltage-current phase angle and impedance, it investigates a data analysis method for fault detection. Lastly, it puts forward a technique to cope with the intricacy and variety of power grid equipment using the CNN model. This technique incorporates an integrated attention mechanism within a one-dimensional CNN model to capture the implicit mapping between voltage, active power, and reactive power at any time in the power system, thereby allowing the model to self-learn multi-device characteristics. The objective of this paper is to extract extensive information about electrical quantities before the occurrence of oscillation and establish threshold values based on typical operating characteristics to assess abnormal patterns, thereby facilitating the early detection of faults and warning quantification.

## Model construction and theoretical analysis

### Stability analysis model of multi-power electronic grid-connected system

The purpose of stability model analysis is to reveal the mechanism and characteristics of harmonic disturbance caused by power electronic grid-connection. Compared with conventional thermal generating units, power electronic equipment has the advantage of fast output response, but its moment of inertia is low, so it needs to be discussed in the stability of grid-connected system^18,19^. This also lays a theoretical foundation for subsequent power output density analysis and moving average method.

The stability of the AC system with multiple DC feeds is mainly affected by the dynamic interaction between the stations at the side of the network through the AC system^4^. The research content includes two aspects: on the one hand, the influence of the dynamic interaction within the two-end HVDC system on the stability of AC system; on the other hand, the influence of the dynamic interaction through the AC network between multiple two-end HVDC systems on the stability of the system. Under the joint action of these two aspects, harmonic disturbance will be produced on the access point^20^.

**Figure 2:**
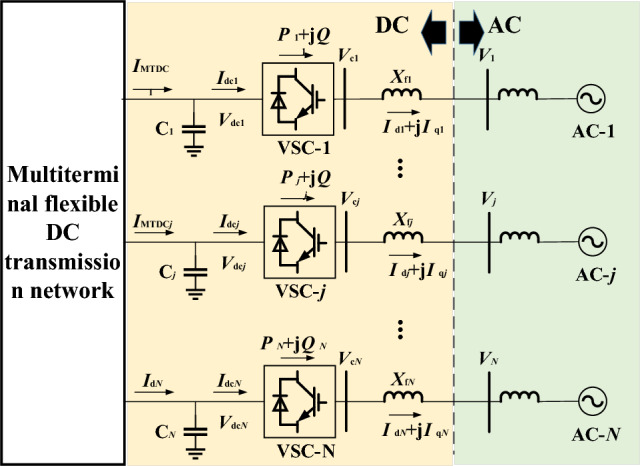
AC power grid with multiple power electronic input.

Figure 2 shows an DC-AC hybrid power system with n-terminal flexible DC grid^21^. In the system, each power electronic device is connected to an independent AC system. $C_{j}$ and $V_{dcj}$ represent DC capacitance value and voltage value of group j power electronic equipment. $I_{dj}$ and $I_{dcj}$ are the input and output current of the capacitor at the DC side of VSC-j. $P_{j}$ and $Q_{j}$ are the active power and reactive power output of VSC-j. $V_{j}$ and $X_{fj}$ are the AC voltage and filter reactance of the AC side of VSC-j. $I_{dj}$ and $I_{qj}$ are d current and q current of the AC side of VSC-j.

Taking the dotted line in Fig. 2 as the boundary, the n-terminal AC-DC hybrid power system is divided into several independent AC subsystems by adopting the block modeling method (AC system, j = 1, 2 … N) and an n-terminal DC subsystem. Then the open loop linearization model is obtained for each subsystem. Finally, the closed-loop linearization model of AC-DC hybrid power system is obtained by splicing multiple open-loop sub-systems with input and output variables as interfaces between subsystems. The details are as follows.

For each independent AC subsystem j in Fig. 2, the output power of VSC-j can be set as $P_{j} + jQ_{j}$. j = 1, 2, … N as the input variable of AC subsystem j, the node voltage $V_{j}$, j = 1, 2 … N as the output variable, the linearized model of AC subsystem j is Eq. 1:

1 $$ \left\{ \begin{gathered} {{d\vartriangle X_{acj} } \mathord{\left/ {\vphantom {{d\vartriangle X_{acj} } {dt}}} \right. \kern-0pt} {dt}} = A_{acj} \vartriangle X_{acj} + b_{pj} \vartriangle P_{j} + b_{qj} \vartriangle Q_{j} \hfill \\ \vartriangle V_{j} = c_{acj}^{T} \vartriangle X_{acj} + d_{pj} \vartriangle P_{j} + d_{qj} \vartriangle Q_{j} \hfill \\ \end{gathered} \right. $$

where $\vartriangle X_{acj}$,j = 1, 2 … N is the vector of all the state variables of AC system j. To arrange Eq. (1) in the form of a transfer function, we have Eq. 2:

2 $$ \left\{ \begin{gathered} \vartriangle V_{j} = g_{pj} (s)\Delta P_{j} + g_{qj} (s)\Delta Q_{j} \hfill \\ g_{pj} (s) = c_{acj}^{T} (sI - A_{acj} )^{ - 1} b_{pj} + d_{pj} \hfill \\ g_{qj} (s) = c_{acj}^{T} (sI - A_{acj} )^{ - 1} b_{qj} + d_{qj} \hfill \\ \end{gathered} \right. $$

Corresponding to the AC subsystems mentioned above, a multi-terminal DC open-loop sub-system can be formed with the voltage amplitude of AC system j as the input variable and the output power $P_{j} + jQ_{j}$ of VSC as the output variable. The following takes master–slave control as an example to discuss how to establish the open loop linearization model of DC energy electronic power grid.

**Figure 3:**
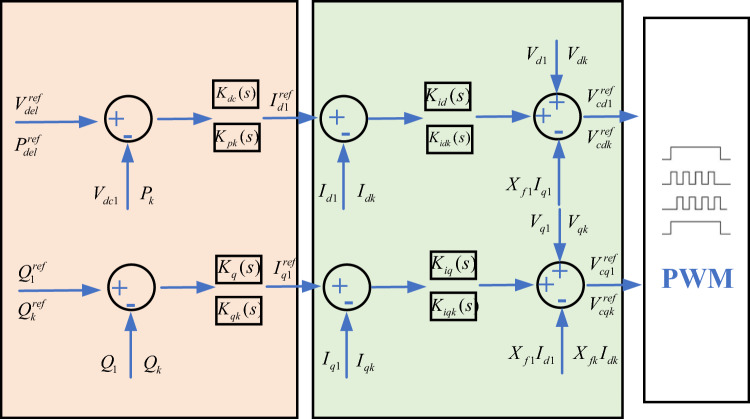
Power electronic control system model.

Assume that in the system shown in Fig. 2, VSC-1 adopts constant DC voltage control, VSC-j adopts constant active power control, and the control system block diagram of converter station is shown in Fig. 3.

The transfer function of each PI controller is Eq. (3)

3 $$ \begin{array}{*{20}r} \hfill {K_{dc} (s) = K_{dcp} + K_{dci} /S} & \hfill {K_{q} (s) = K_{qp1} + K_{qi1} /S} \\ \hfill {K_{id} (s) = K_{idp1} + K_{idi1} /S} & \hfill {K_{iq} (s) = K_{iqp1} + K_{iqi1} /S} \\ \hfill {K_{pk} (s) = K_{ppk} + K_{pik} /S} & \hfill {K_{qk} (s) = K_{qpk} + K_{qik} /S} \\ \hfill {K_{idk} (s) = K_{idpk} + K_{idik} /S} & \hfill {K_{iqk} (s) = K_{iqpk} + K_{iqik} /S} \\ \end{array} $$

Assume that all PWM algorithms are steady-state adjusted, which means, the following Eq. (4) is satisfied:

4 $$ \left\{ \begin{gathered} V_{cdj} = V_{cdj}^{ref} \hfill \\ V_{cqj} = V_{cqj}^{ref} \hfill \\ \end{gathered} \right. $$

Equation (5) of the transfer function can be constructed according to the above derivation:

5 $$ \begin{gathered} \Delta I_{d1}^{ref} = (K_{dcp} S + K_{dci} ) * \frac{{\Delta V_{dc1} }}{S} \hfill \\ \Delta I_{dk}^{ref} = (K_{ppk} S + K_{pik} ) * \frac{{\Delta P_{k} }}{S} \hfill \\ \Delta I_{qk}^{ref} = (K_{qpk} S + K_{qik} ) * \frac{{\Delta Q_{k} }}{S} \hfill \\ \end{gathered} $$

Further, Eq. (6) of the transfer function of voltage control and active power control can be written:

6 $$ \begin{gathered} \Delta I_{d1} = G_{p1} (s)\Delta V_{dc1} \hfill \\ \Delta I_{q1} = G_{q1} (s)\Delta Q_{1} \hfill \\ \Delta I_{dj} = G_{pj} (s)\Delta P_{j} \hfill \\ \Delta I_{qj} = G_{qj} (s)\Delta Q_{j} \hfill \\ \end{gathered} $$

where, for j = 1,2,…,N, we have the Eq. (7)

7 $$ \begin{gathered} G_{pj} (s) = [\omega_{0} K_{id} (s)K_{dc} (s)]/[SX_{fj} + \omega_{0} K_{id} (s)] \hfill \\ G_{qj} (s) = [\omega_{0} K_{iq} (s)K_{q} (s)]/[SX_{fj} + \omega_{0} K_{iq} (s)] \hfill \\ \end{gathered} $$

Suppose that in power electronic equipment, phase-locked loops are ideal, and the direction of $V_{j}$ is selected as positive, which means, in the D-q coordinate system, recombination with axis d, then the linear equation of output power $P_{j} + jQ_{j}$ and I, in j = 1, 2 … N, can be expressed as Eq. (8):

8 $$ \begin{gathered} \Delta P_{j} = I_{dj0} \Delta V_{j} + V_{j0} \Delta I_{dj} = V_{dcj0} \Delta I_{dcj} + I_{dcjo} \Delta V_{dcj} \hfill \\ \Delta Q_{j} = - I_{qj0} \Delta V_{j} - V_{j0} \Delta I_{dj} \hfill \\ \end{gathered} $$

Considering the DC convergent transmission part of new energy, Eq. (9) is given

9 $$ P_{j} = V_{dcj} I_{dcj} = V_{dcj} ( - C_{j} {\raise0.7ex\hbox{${dV_{dcj} }$} \!\mathord{\left/ {\vphantom {{dV_{dcj} } {dt}}}\right.\kern-0pt} \!\lower0.7ex\hbox{${dt}$}} + I_{{MTDC_{j} }} ) $$

where the derivative of $I_{{MTDC_{j} }}$ in time satisfies the following Eq. (10):

10 $$ dI_{{MTDC_{j} }} /dt = \sum\nolimits_{i = 1}^{n} {y_{ji} V_{dci} } $$

According to Eq. (9), the matrix equation can be obtained .As shown in Eq. (11):

11 $$ \left[ \begin{gathered} \Delta P_{1} \hfill \\ \Delta P_{j} \hfill \\ \end{gathered} \right] = W(s)\left[ \begin{gathered} \Delta V_{dc1} \hfill \\ \Delta V_{dcj} \hfill \\ \end{gathered} \right] + U\left[ \begin{gathered} I_{{MTDC_{1} }} \hfill \\ I_{{MTDC_{j} }} \hfill \\ \end{gathered} \right] $$

According to Eq. (10), we can get Eq. (12):

12 $$ S\left[ \begin{gathered} I_{{MTDC_{1} }} \hfill \\ I_{{MTDC_{j} }} \hfill \\ \end{gathered} \right] = Y(s)\left[ \begin{gathered} \Delta V_{dc1} \hfill \\ \Delta V_{dcj} \hfill \\ \end{gathered} \right] $$

where $Y(s)$ is the admittance matrix of the new energy DC convergence part, and the value range of j is j = 2, 3 …, N, from this we further obtain the relationship between *W(s)*, *U*, *V*, and *I* as shown in Eqs. (13) and (14).

13 $$ W(s) = - \left[ {\begin{array}{*{20}c} {I_{dc10} + sC_{1} V_{dc10} } & 0 & \cdots & 0 \\ 0 & {I_{dc20} + sC_{2} V_{dc20} } & \cdots & 0 \\ \vdots & \vdots & {} & \vdots \\ 0 & 0 & 0 & {I_{dcN0} + sC_{2} V_{dcN0} } \\ \end{array} } \right] $$

14 $$ \begin{gathered} \hfill \\ U = - \left[ {\begin{array}{*{20}c} {V_{dc10} } & {} & {} & {} \\ {} & {V_{dc20} } & {} & {} \\ {} & {} & \ddots & {} \\ {} & {} & {} & {V_{dcN0} } \\ \end{array} } \right] \hfill \\ \end{gathered} $$

Finally, we built a mapping model between new energy DC voltage and output active power as Eq. (15):

15 $$ \Delta V_{dvi} = \sum\nolimits_{i = 1}^{n} {f_{li} \Delta P_{i} } $$

Through the derivation in this section, we obtained the stability analysis model of the multi-power electronic grid-connected system. The model can be inferred as follows:

- Under the ideal state, there is no harmonic disturbance in the output power P, but when the new energy faces the fluctuations of the input side, such as wind, light intensity, etc., the output power P will fluctuate;
- There is no inertia link between input and output, which means, in the process of load fluctuation, new energy does not have self-regulation ability, and can not be adjusted by the excitation or rotation of thermal power units to achieve frequency stability;
- In case of internal failure of new energy, the output work will drop rapidly, and there is no work angle curve of conventional thermal power units. The static and transient stability of frequency and voltage is insufficient;
- New energy fluctuated rapidly and belongs to linear conduction, so the impact brought by its fluctuation was more obvious than the load fluctuation in the time domain.

### New energy disturbance energy characteristic analysis method based on density curve

**Figure 4:**
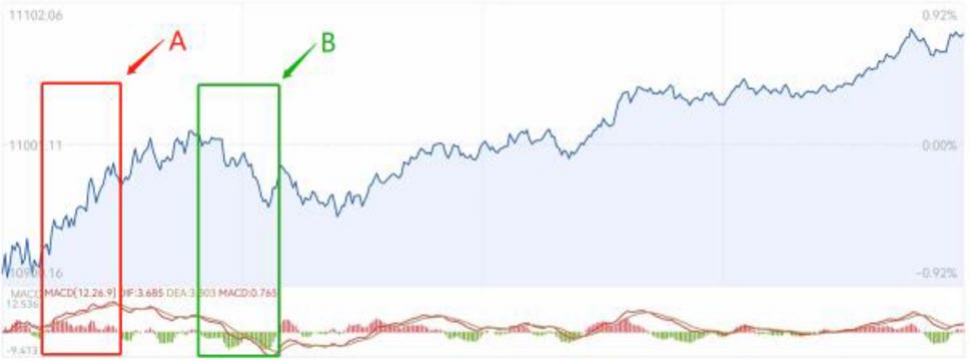
Graph of the relationship between capital volume and price.

The previous chapter deduces the potential power disturbance risk of new energy grid connection. In this chapter, a feature analysis method based on disturbance energy density is constructed. Different from the threshold determination method adopted by current scholars, we choose disturbance energy density as the target reference, which can reflect the change of disturbance trend^22^. The basic idea is similar to the amount of capital in the stock trading system, as shown in Fig. 4:

This is a graph of the relationship between the index value and the amount of funds in the NASDAQ index over a period. In region A, intensive buying funds indicate the rising trend of stock prices, while the result of capital outflow is largely reflected in the decline of the index, as shown in region B in Fig. 4.

From this we can construct concept maps. In the power system, the sudden increase of disturbance can be considered as a kind of capital injection. The increase of disturbance energy area per unit time can be regarded as a continuous capital injection, which can reflect the precursor of oscillation. The perturbation power density is calculated as Eq. (16):

16 $$ \rho = \frac{{\sum\limits_{{t_{start} }}^{{t_{end} }} {\sum\limits_{i = 1}^{i = 5} {W_{i} } } }}{{t_{end} - t_{start} }} $$

**Figure 5:**
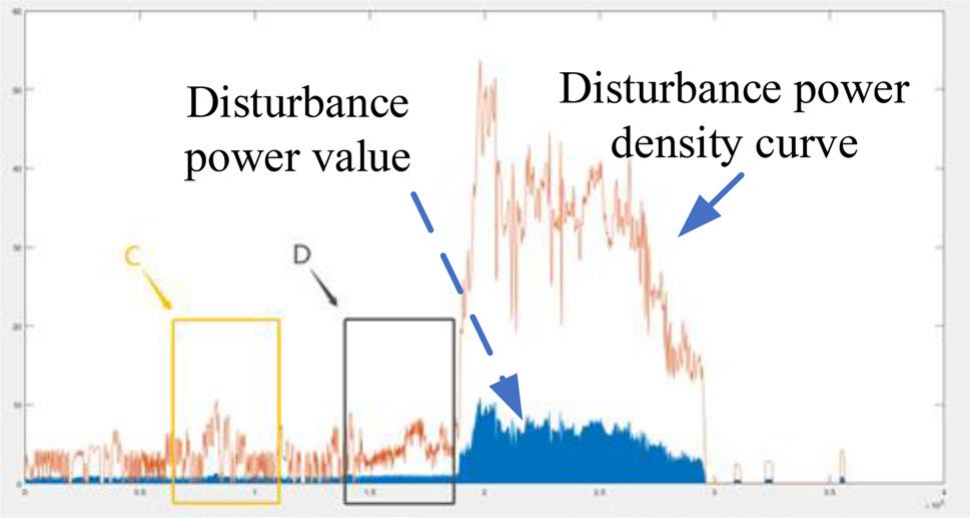
Diagram of disturbance power density and oscillation.

In Formula 16, $t_{start}$ and $t_{end}$ are the division on a time scale, because the calculation range of power density is between two specific time points. The selection of specific scope should be combined with the sensitivity requirements of the system. If high dynamic data judgment is needed, the time scale is generally 2–5 s. If the system itself is robust, the time scale can be extended to 10 s or even higher. The determination of robustness will be elaborated in the following Sect. "Analysis method of voltage and current Angle and impedance characteristics of new energy based on equal-line theory". Wi is the power amplitude of the top 5 disturbance components. Here, Fig. 5 is taken as an example to illustrate the relationship between the power density of disturbance and the precursor of oscillation.

The data in Fig. 5 came from the PMU device, and the interval of data points was 20 ms. According to Eq. (16), we calculated the disturbance energy every 5 s to construct the orange disturbance power density curve in the figure. Combined with the blue perturbation energy amplitude, we focus on regions C and D. In region C, we see that the disturbance power density curve rises, but the fluctuation is obvious, and there is no trend of continuous enhancement. However, in region D, there is no obvious tendency for the curve to recalibrate, so there is a significant subsequent oscillation. Formula 17 can be used to describe the trend of the power curve:

17 $$ \rho_{n} = \frac{{\sum\limits_{i = 1}^{i = n} {\rho_{i} } }}{n} $$

In Eq. (17), if n is 5, 10, 15, then, $\overline{\rho }_{5}$, $\overline{\rho }_{10}$, $\overline{\rho }_{15}$ respectively represent the mean values of disturbance power curves at 5, 10, and 15 unit statistical scales. As shown in Eq. (18) of the relationship

18 $$ \left\{ \begin{gathered} \overline{\rho }_{5} > \overline{\rho }_{10} > \overline{\rho }_{15} \begin{array}{*{20}c} {} & {} & {} \\ \end{array} {\text{Upward trend}} \hfill \\ \overline{\rho }_{15} > \overline{\rho }_{10} > \overline{\rho }_{5} \begin{array}{*{20}c} {} & {} & {} \\ \end{array} {\text{Downward trend}} \hfill \\ other \hfill \\ \end{gathered} \right. $$

If the moving average of 5 is greater than the moving average of 10 and is greater than the moving average of 15, we believe that it is a "multi-sided" arrangement, and the disturbance power density presents an obvious upward trend. If the moving average of 15 is greater than the moving average of 10, also is greater than the moving average of 5, we consider it to be a "short" arrangement, with density trending down. Region D obviously conforms negatively to the "multi-sided" trend, while region C is disorganized and belongs to the other category, which cannot be used for trend judgment.

### Analysis method of voltage and current Angle and impedance characteristics of new energy based on equal-line theory

The angle between voltage and current of stationary load should be stable. Grid-connected systems of photovoltaic, direct drive fan and other new energy sources, as well as flexible DC power transmission can be regarded as converter grid-connected models, which have multi-time scale characteristics, and their instability phenomenon shows broad frequency characteristics^23–25^. The main criteria for taking phase locked loop as the leading link include: stability criteria for extracting the main loop of phase locked loop; Generalized impedance criterion of phase angle impedance is extracted. Generalized torque coefficient criterion of electrical inertia is extracted, etc. In this paper, this kind of stability problem is called "virtual work Angle" stability. The calculation method of voltage and current Angle is shown in Formula 19:

19 $$ \phi_{u - i}^{n} = \phi_{u}^{n} - \phi_{i}^{n} $$

**Figure. 6:**
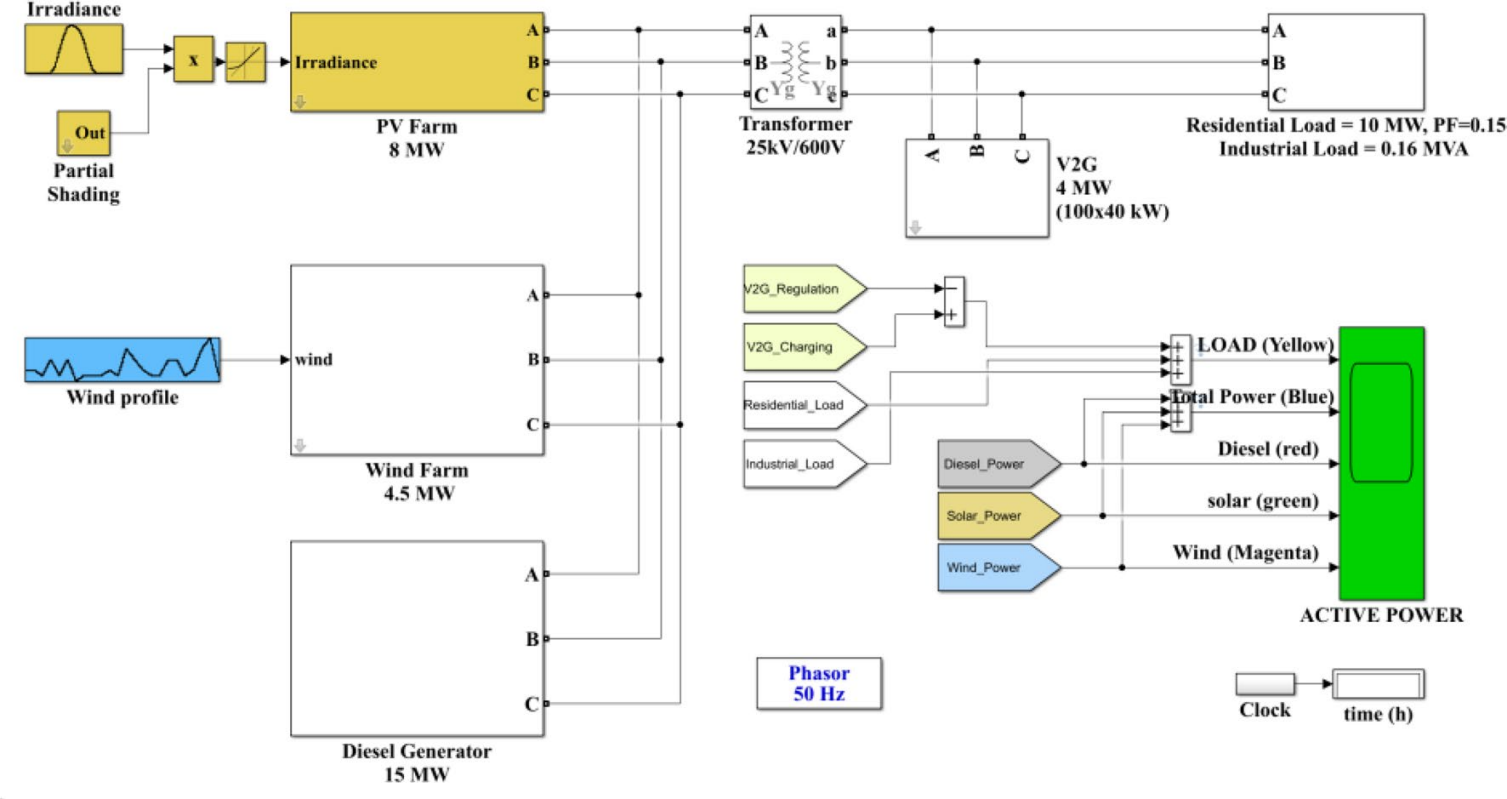
Simulation of Angle fluctuation of new energy grid connection.

**Figure 7:**
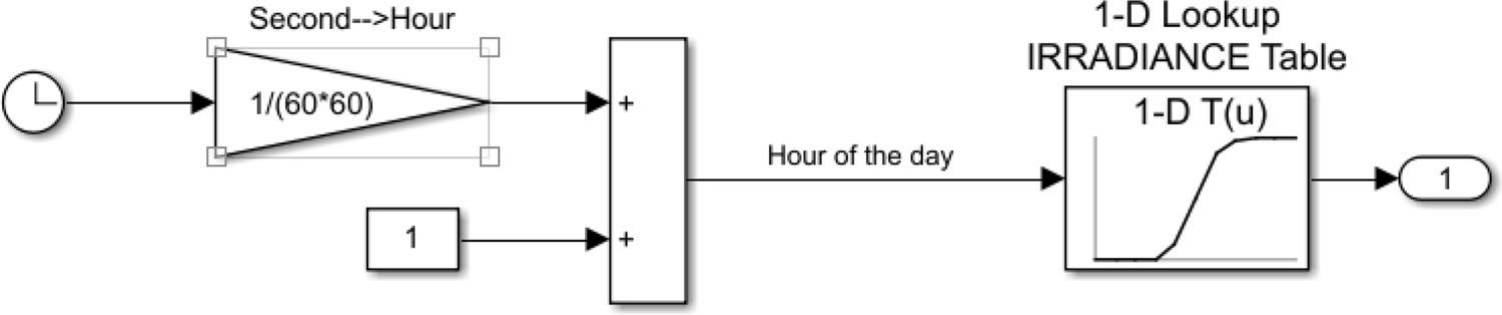
Second level illumination simulation model.

**Figure 8:**
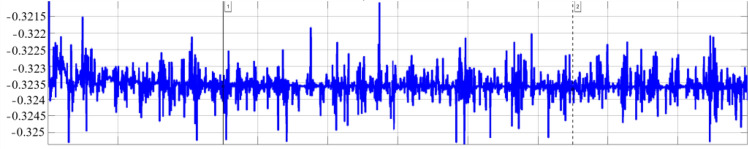
A-phase voltage-current Angle difference at junction point.

In order to better reflect that familiarity with the angle between voltage and current is an important feature of new energy junction points, we built a simulation model. The architecture is shown in Fig. 6:

For the new energy groups in the simulation, we construct grid-connected scenes of photovoltaic, wind power and other typical new energy at the source end^26,27^. In the input parameters of photovoltaic, we built varying illuminance to simulate complex lighting scenes in the real situation. The model is shown in Fig. 7:

Similarly, on the input side of the fan, we also introduced the changing wind speed by imitating the light. At the junction point of new energy, according to model 11 and formula 14, the angle fluctuation of voltage and current can be observed, as shown in Fig. 8:

Figure 8 reflects the following fluctuations of the phase angle under the fluctuation of new energy. Theoretically, if the electricity volume is described according to formula 20, the change trend of phase angle will be easier to understand under the condition of sharp fluctuations of new energy output.

20 $$ Y(t) = y(t)e^{j[\omega (t)t + \varphi (t)]} $$

Different from the fixed initial phase angle expression $\varphi (t)$, formula 15 also constructs the phase angle relation $\omega (t)t$,which changes with time, which is very suitable for the electrical vector description in the scenario of load new energy fluctuation.

**Figure 9:**
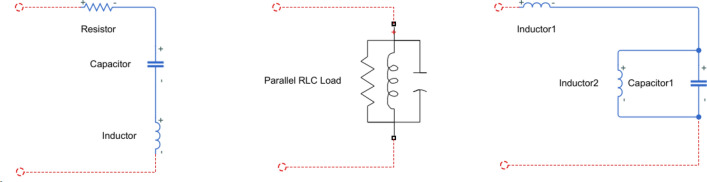
Equivalent model of power network oscillating circuit.

Impedance is a key factor in the analysis of power grid, and it has the highest advance in the prediction of oscillation. In the control theory, there is usually the concept of damping, whose magnitude is generally related to the stability of the system. It refers to the performance of the system that recovers from the initial deviation state to the original equilibrium state after the disturbance disappears. In the power system, the disturbance power can be regarded as a kind of burst excitation. Whether the power system can absorb the disturbance and maintain its own stability is the key to the grid connection of new energy^28,29^. However, the impedance of the power system is not constant, as shown in Fig. 9:

Figure 9 reflects two key concepts. The first is the equivalence of two ports. Observing the power grid from the measuring point, the measured power grid can form a two-port network. The second is the natural oscillation frequency. Since the two-port network is composed of series and parallel RLC, there must be a resonant frequency point. In power system planning, our design concept is to make the grid work frequency as far away from the resonance point as possible. However, the new energy grid has the characteristics of wide frequency domain, which makes the disturbance spectrum of the grid widely distributed. The power electronic equipment on the load side makes the RLC equivalent circuit complex and changeable. The interaction between the source end and the load side increases the probability of oscillation risk in the power network.

**Figure 10:**
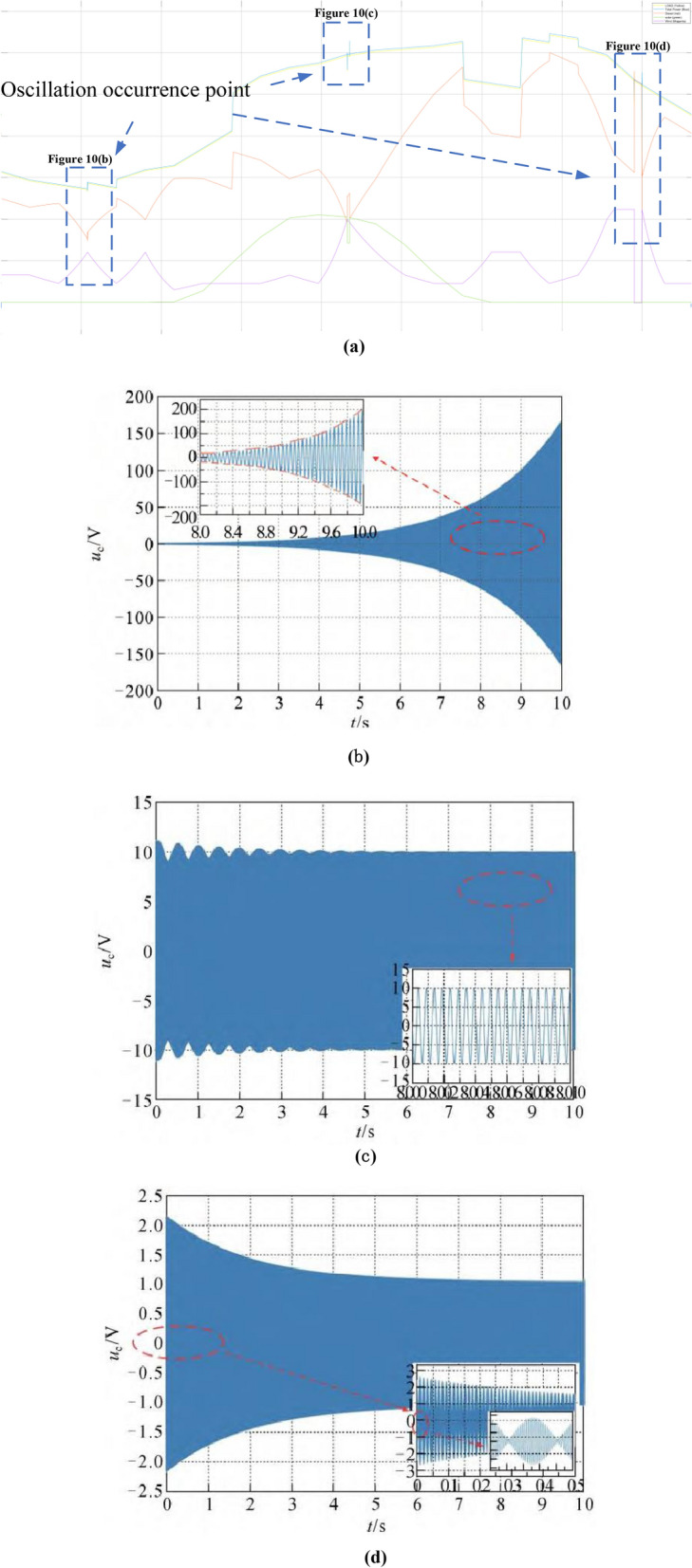
(a) Oscillatory waveform caused by adjusting impedance. (b) Under damped, the oscillations do not converge. (c) Critically damped, oscillations do not diverge. (d) Over damped, the oscillation converges too much.

Based on the simulation model in Fig. 6, we simulate three oscillations according to possible resonant frequency points. As shown in the dashed box in Fig. 10, due to the mismatch between the instantaneous impedance of the power supply and the grid, oscillations with different characteristics are triggered, including divergence, holding and convergence. The specific matching process can be described by Formula 21:

21 $$ {\text{USI}}_{1} = \rho [G_{g}^{ - 1} (s)G_{s}^{ - 1} (s)|_{s = j\omega ,\omega > 0} ] $$

where, G_g_(s) is the transfer function equation of power electronic power supply station; G_s_(s) is the transfer function equation of AC power grid; Represents the spectral radius of the calculation of the S-domain equation. The dynamic index USI_1_ in this paper considers the dynamic characteristics of power electronic power supply and AC power network, as well as the dynamic behavior of power electronic power supply and its control system, to obtain more accurate and effective safety and stability risk assessment results of power electronic power system.

### Safety margin evaluation algorithm of fusion attention mechanism and one-dimensional convolutional neural network

The prediction of power network oscillation risk in this paper is based on the discrimination of normal state, and the abnormal state is identified based on normal state. However, in the actual situation, there is a large amount of node information in the power system. Due to different voltage levels and different equipment types, the range of the positive normal is also different. It is necessary to train the neural network to identify the approximate positive normal range of each device. A certain clustering algorithm is needed to aggregate devices of different voltage levels in a normal threshold prefabricated range, to realize the discrimination of abnormal states.

**Figure 11:**
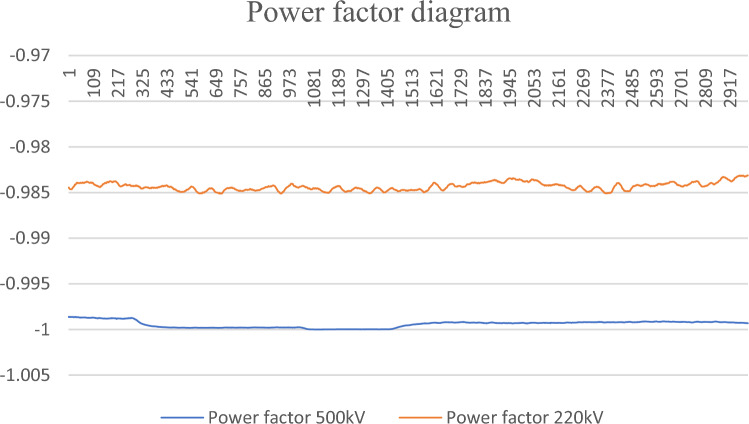
Comparison of 220 kV and 500 kV power factor curves.

Before explaining the basic theory of neural networks, this paper will explain the limitations of the general trend statistical theory mentioned in Eq. (18) through two typical monitoring data of different voltage levels, and explain the necessity of data cluster analysis under different voltage levels and different load scenarios in the power system. As shown in Fig. 11:

In Fig. 11, the power factor curves of two power transformers with different voltage levels are shown. First of all, both transformers did not oscillate during the period when the data was displayed, but we can see that the power factor curves of 220 kV transformers fluctuated dramatically. If a unified evaluation standard is used to evaluate 220 kV on the basis of 500 kV, it will inevitably lead to 220 kV being wrongly judged as having the risk of oscillation. On the contrary, if 220 kV is used as the basis to evaluate 500 kV, the 500 kV threshold will be too large and the risk calculation will be insensitive.

The data set obtained from transient power Angle and transient voltage simulation is $X = \{ X_{1} ,X_{2} ,...,X_{m} \} ,X \in {\mathbb{R}}^{t*m}$, t is the length of time series data and m is the number of buses monitored. First, given query vector $O_{i}$ to query information in data set X. The dot product model is used to calculate the attention score of each input vector, as shown in Eq. (22):

22 $$ O_{i} = score(X_{i} ,q) = X_{i}^{T} q $$

where i ∈ [1, M] is a sequence position of input and output vectors.

The obtained attention score $O_{i}$ is normalized, weighted, and summed with the corresponding score $X_{i}$, and the calculation result is output, as shown in Eq. (23:

23 $$ result = \sum\nolimits_{i = 1}^{m} {\max [O_{i} ]} X_{i} $$

1D-CNN performs deep extraction of input features through multi-layer convolution and pooling layers ^30–32^. Its convolution core is a weight matrix. During the calculation process, important features in the input data are extracted through convolution checking of local regions to reduce the computational complexity through weight sharing. The convolution operation process is as follows Eq. (24):

24 $$ \left\{ \begin{gathered} x_{m}^{n} = \sum\nolimits_{i = 1}^{{N_{n - 1} }} {(w_{im}^{n - 1} \otimes s_{i}^{n - 1} )} + b_{m}^{n} \hfill \\ y_{m}^{n} = \sigma (x_{m}^{n} ) \hfill \\ \end{gathered} \right. $$

In Eq. (24), $x_{m}^{n}$ represents the m-th input of layer n in the network, $s_{i}^{n - 1}$ represents the i-th output of layer n-1, $w_{im}^{n - 1}$ represents the convolution kernel of the corresponding channel $s_{i}^{n - 1}$, $N_{n - 1}$ is the number of outputs $s_{i}^{n - 1}$ of layer n-1. $\otimes$ represents the convolution operation, $\otimes$ represents the deviation of the m-th output, $\sigma (x_{m}^{n} )$ represents the activation function, and $y_{m}^{n}$ represents the output result.

After the convolutional pooling operation, the network enters the full connection layer, and then outputs the result. In regression problems, the network generally uses the Mean Squared Error (MSE) function to calculate losses. The calculation results of the loss function are used to reverse guide the updating of parameters at each layer of 1D-CNN, and ultimately complete the training.

**Figure 12:**
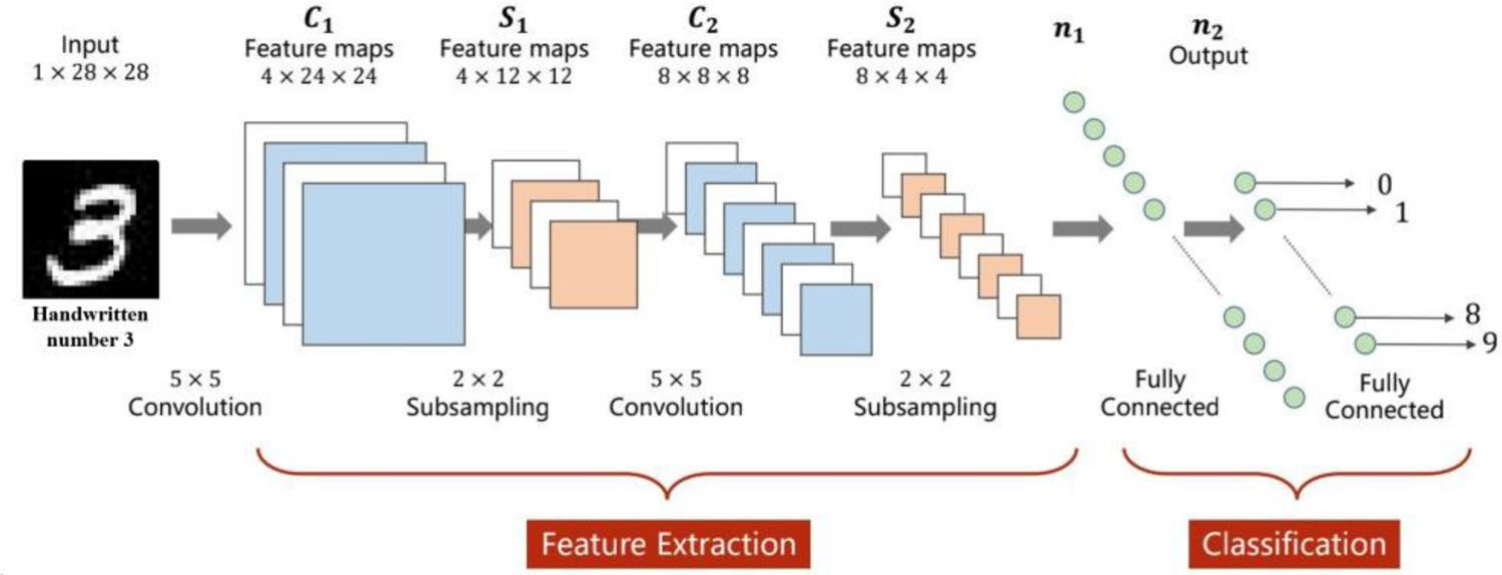
Image neural network recognition process.

Before introducing power grid data to CNN, the paper compares the class to easily understood image recognition, explaining what neural networks train in power systems and how they support weak point recognition, as shown in Fig. 12.

In image processing, in order to simulate pixel changes in images in the field of vision, commonly known as changes in image size caused by changes in distance, image compression training is required. The features in the image are contained in the pixels, and the full connection of image features at different resolutions is also different. The purpose of training is to identify the key information of the same image at different resolutions. Many scholars have begun to apply image processing concepts such as CNN to power system analysis. In reference^37^, Feng et al. proposed that due to its low dependence on system models, strong learning capabilities for nonlinear and complex relationships between large amounts of data, and rapid adaptability to time-varying environments, is helpful in solving wide-band oscillations problems.

The technical relevance of power grid measurement data and image recognition will be described in Table 1 below:

**Table 1:** Comparative relationship between image recognition and power grid feature recognition.

	Image recognition	Grid measurement data
Compression and segmentation	Compress images at different resolutions and divide them into N * N regions to find key features	There is also a need for compression and segmentation of power grid measurement data on a time scale. Different disturbances have different durations and frequencies in the time dimension, requiring segmentation to explore feature correlation relationships ^33^
Feature extraction	It is a gradient relationship in the image	In the power grid, it is the time domain characteristic, slope characteristic, and mutation characteristic of the monitoring quantity
Classification	Distinguish between the background and the target	Finding fault data fragments in massive monitoring data

### Technical route for oscillation risk assessment

**Figure 13:**
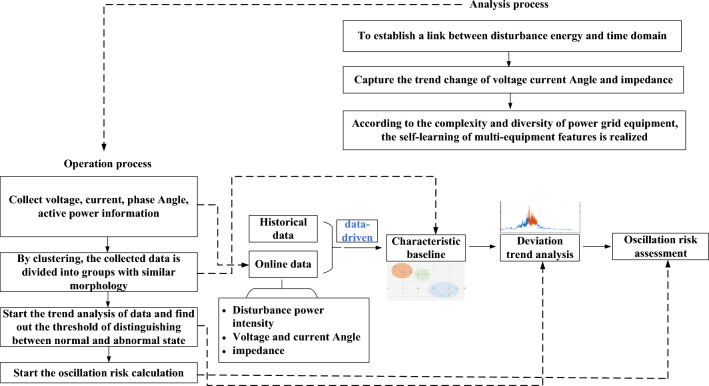
Risk assessment technical route.

As shown in Fig. 13, this paper forms window feature identification of oscillation data according to historical oscillation data. By means of data statistics and inference, the window characteristic factors were extracted to form the factors of oscillation risk assessment based on the disturbance power density change, harmonic power growth trend, harmonic frequency distribution change, voltage and current phase angle change trend, etc., and the weight ratio was adopted to form an overall oscillation early warning strategy that integrated all factors.

### Technical route for identifying vulnerabilities

**Figure 14:**
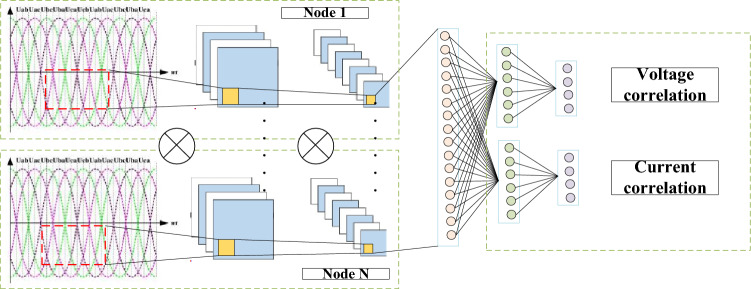
Structure of one-dimensional convolutional neural network model.

Based on the characteristics of power system data, the research adopts a one-dimensional convolutional neural network model structure incorporating attention mechanisms (as shown in Fig. 14). When predicting transient power angles and transient voltages in the research, the U/P/Q of the load bus is used as the input feature. During the learning process, the U/P/Q of different load buses are first scanned separately, and the specific process is to first reorganize the U/P/Q data of each bus at all times into a one-dimensional vector, In order to preserve the timing information at different times, 1D-CNN with a convolution kernel size of 1 is used to convolution the reconstructed data. The above convolution method enables the extracted features of 1D-CNN to be the relationship characteristics between the corresponding U/P/Q of each bus, and can construct an implicit relationship map between voltage, active power, and reactive power at any time. After two layers of convolution, the extracted different bus features are connected to the attention network model. After learning and iterating the weights of the model, different weights can be calculated for each bus-bar feature, thereby obtaining electrical quantity relationships, providing a basis for weak point clustering analysis^34–36^.

## Theoretical applicability analysis

### Verification of perturbation power density method

**Figure 15:**
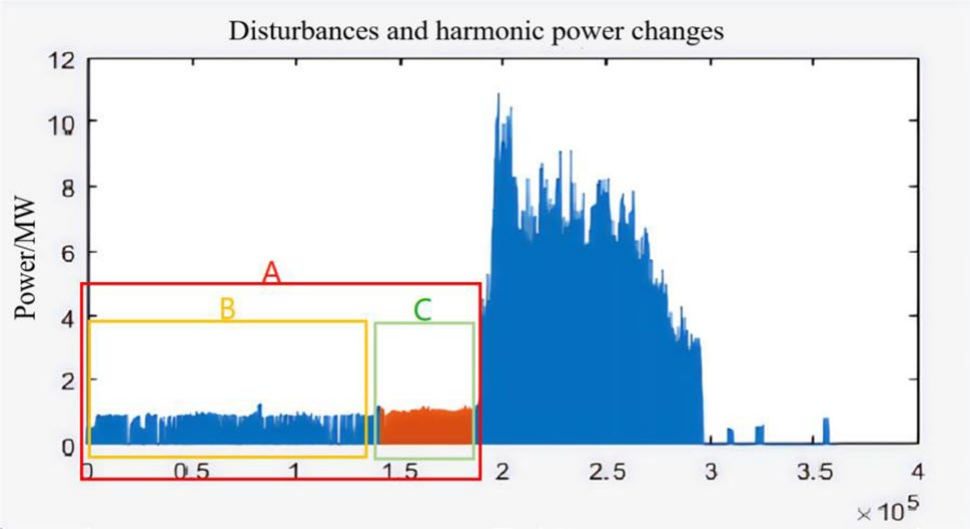
Power diagram of oscillation case.

**Figure 16:**
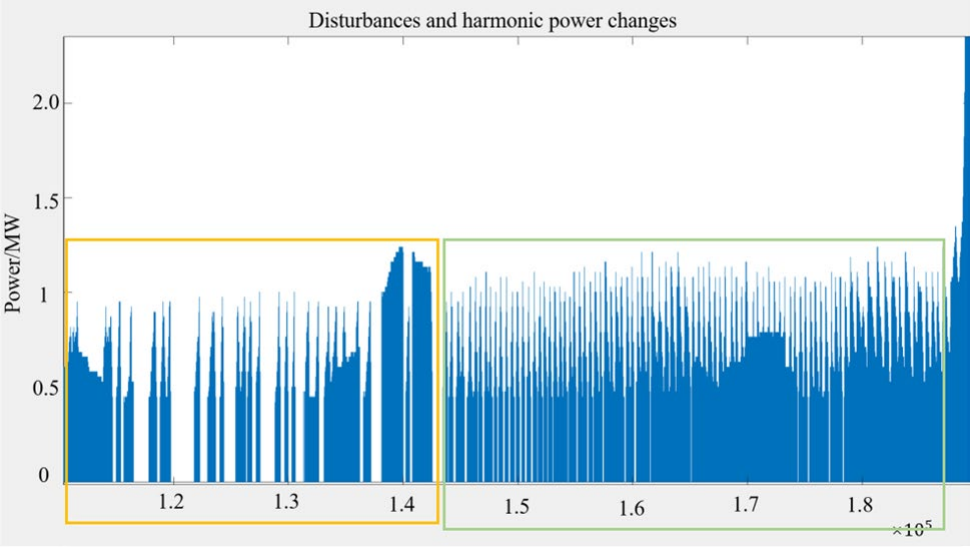
Perturbation power local refinement diagram.

This case is the first harmonic oscillation of Guyuan Wind Farm in China, and the data is collected by PMU device, as shown in Fig. 15:

The horizontal coordinate of the data graph is the serial number of the monitoring data. The interval of each data message is 10 ms. In Guyuan case, the main disturbance components are 40 Hz and 100 Hz. The region A is the pre-oscillation window period. In the whole-time window of region A, the disturbance frequency component always exists, and so does the disturbance power. Therefore, it is impossible to accurately predict the oscillation by relying solely on the statistics of disturbance power. However, when time window A is divided into time window B and C, we find that the intensity of disturbance power in window C is much greater than that in window B. The local comparison is shown in Fig. 16:

It can be seen from Fig. 16 that before the oscillation, the intensity of power disturbance presents an obvious statistical rule, and the intensity tends to strengthen. This verifies the inference of power density in Sect. "New energy disturbance energy characteristic analysis method based on density curve" and the inference of new energy stability in Sect. "Stability analysis model of multi-power electronic grid-connected system".

**Figure 17:**
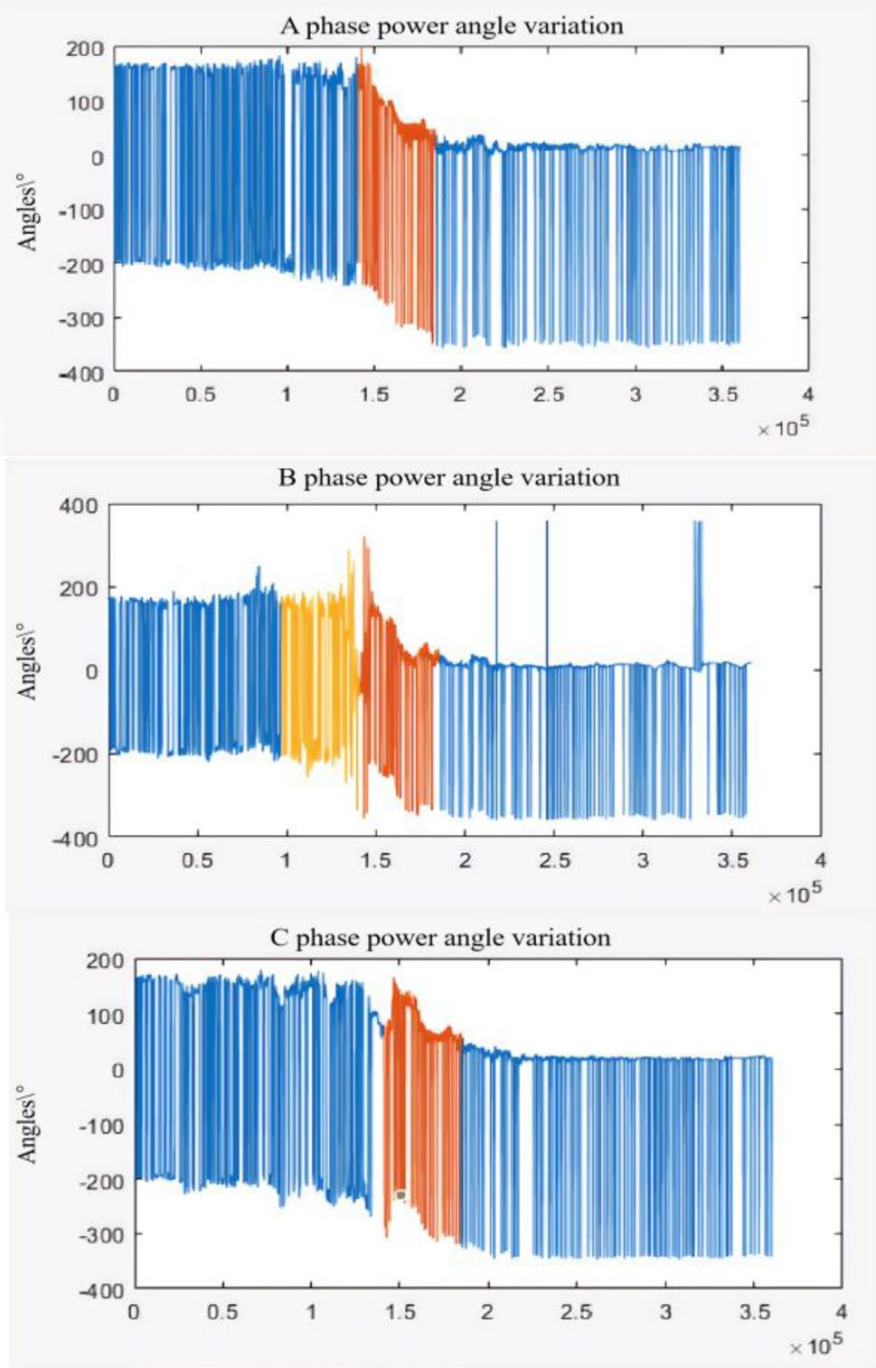
Measured diagram of phase Angle change.

**Figure 18:**
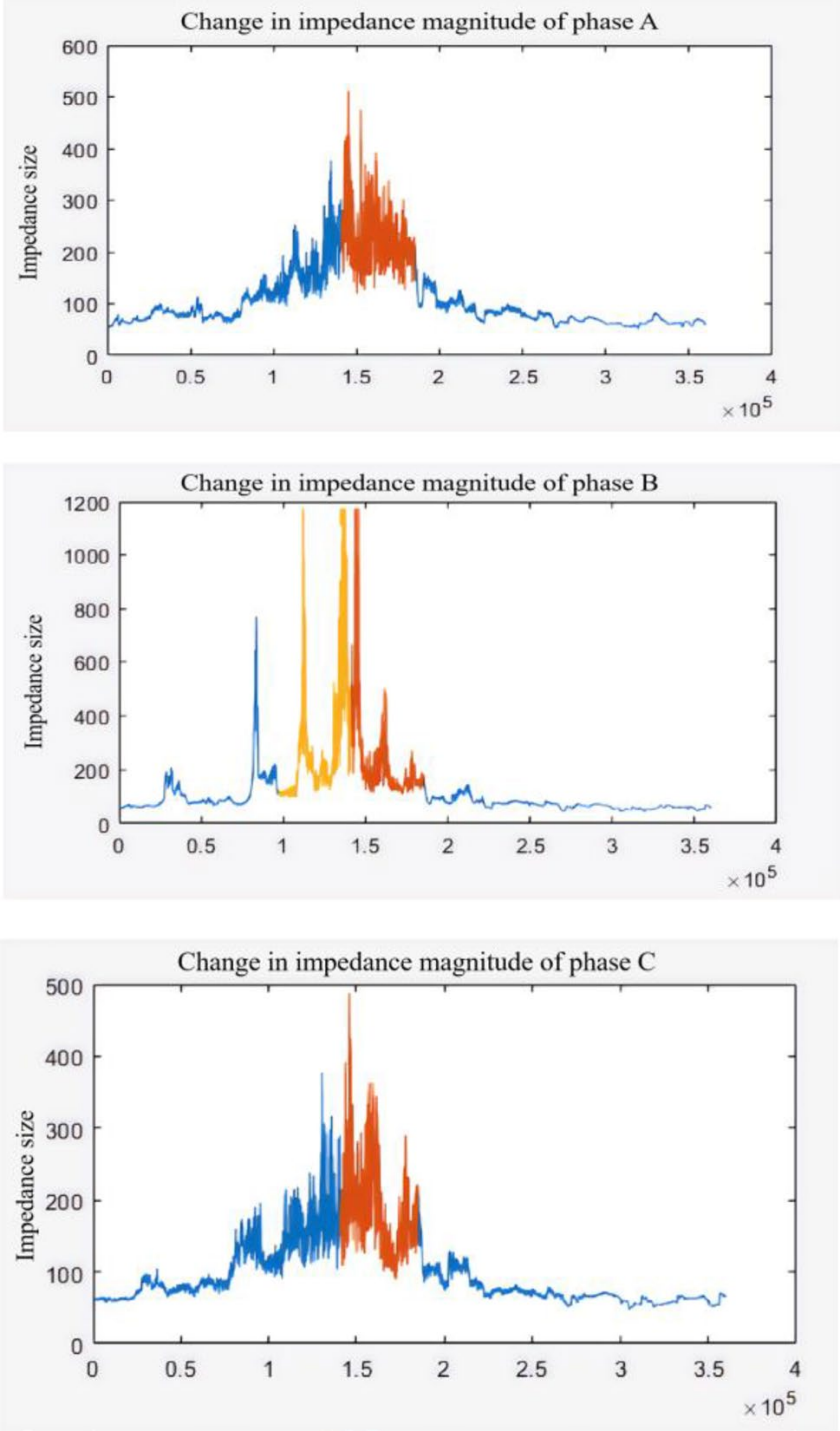
Comparison of impedance and phase Angle trends.

However, it is difficult to select statistical base for the mathematical statistics of power disturbance density, which requires a long time of sampling data to quantitatively define "dense" and "sparse". In order to further explore other electrical characteristics of region C in Fig. 15, we comprehensively compare the variation trend of voltage and current phase angle of A, B and C, as shown in Fig. 17:

Figure 17 calculates the angle between voltage and current of each phase. In the orange marked area, mutations are obviously found, which are highly overlapped with the time of window C in Fig. 15. Thus, it can be inferred that in the case of Guyuan secondary synchronization, the voltage-current angle is an obvious characteristic of electrical volume, and the time of orange marked area is up to 8 min. Therefore, combined with intensive statistics of power disturbance, risk prediction can be effectively realized in advance. Before the orange interval in Fig. 17, we found that phase angle had obvious jitter before the significant trend change. Therefore, we further analyzed the change of damping, as shown in Fig. 18:

The time window of drastic impedance changes overlaps highly with the changes in voltage-current angle. It is obvious that impedance parameters have fluctuated significantly in the jitter stage before the angle trend change. Therefore, the Guyuan case shows that the risk can be estimated 2 ~ 3 min in advance by combining impedance change analysis.

### The moving average method identifies the disturbance precursor

**Figure 19:**
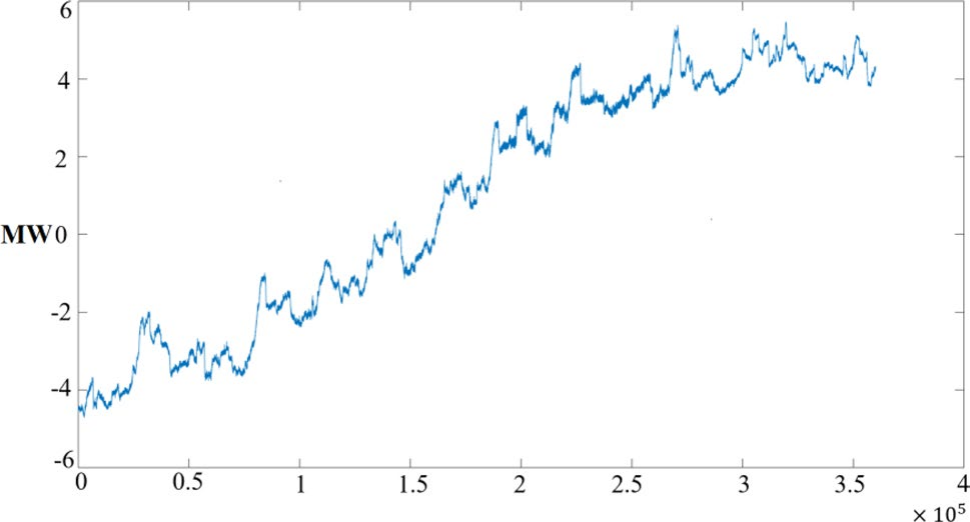
Active power curve with disturbance energy.

At the WAMS station, many data channel resources are sacrificed to obtain the data shown in Fig. 16. In most cases, we can only get the active power curve as shown in Fig. 19. Identifying precursors of disturbances from individual power curves is very challenging. Here we will test the applicability of the moving average theory mentioned in Sect. "New energy disturbance energy characteristic analysis method based on density curve".

From the point of view of power system, the detected power components can be divided into load power and disturbance power. Compared with the load power, the disturbance power component is smaller, the frequency is higher, and the consistency is difficult to capture. From the point of view of increasing disturbance density before the oscillation, the continuous accumulation of disturbance leads to more serious oscillation phenomenon. Taking the relationship between the amount of capital and the index value shown in Fig. 4 as an example, because of the irregularity of capital injection under large market conditions, the average line method can better excavate the effect intensity of small disturbance force.

**Figure 20:**
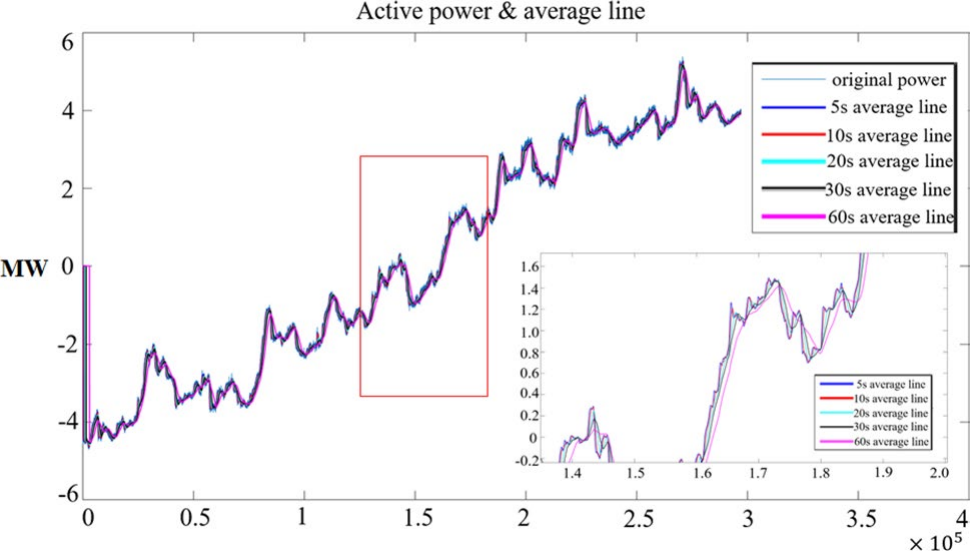
Linear variation trend of average active power.

Therefore, we construct the mean line of the active power curve according to Formula 18, and first discuss the moving averages of 5, 10 and 15. As shown in Fig. 20:

In Fig. 20, we can see that when the load stability disturbance is small, the moving average statistics of different time scales are stable and similar. If the disturbance power changes violently, the moving averages on short time scales will fluctuate violently and often cross with other long time moving averages. To some extent, this reflects the difference between load and disturbance accumulation.

**Figure 21:**
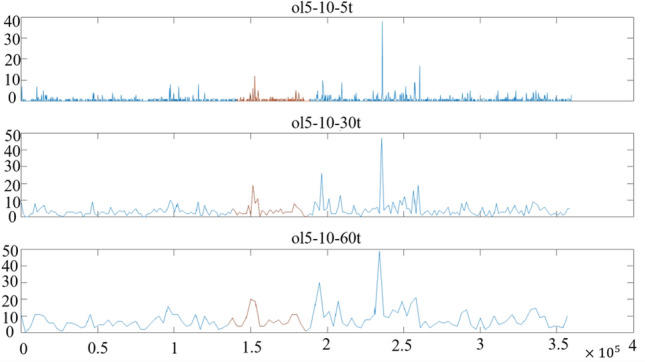
Statistical chart of active power moving average crossing trend.

By further analyzing the statistics of crossing of moving averages, Fig. 21 can be obtained.

As can be seen from Fig. 21, statistical results at different time scales show significant changes in the number of intersections in the oscillation stage (marked in the red box). There are also differences in the statistical properties of the continuous dense state of perturbation before the oscillation (red line). The statistical characteristics are closely related to the power disturbance. However, the situation is slightly different when different moving average data or statistical models with different time scales are selected, but both can well reflect the perturbation characteristics of the oscillations.

### Identification of associated weaknesses

**Figure 22:**
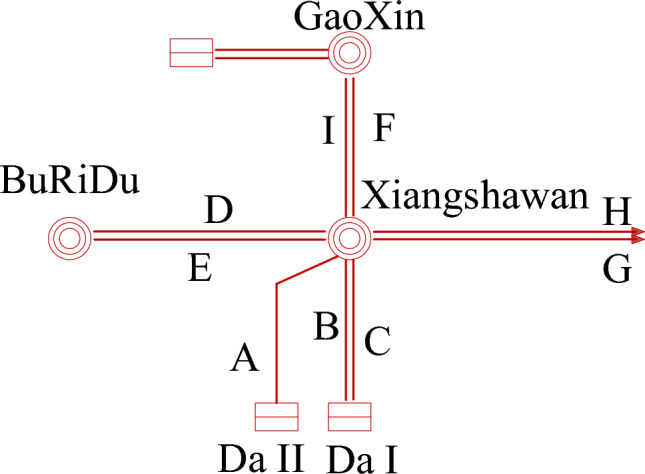
Partial Power Supply Section of North China Power Grid.

In this chapter, we will take the power supply section from Hohhot to Baotou of the North China Power Grid as an example to study the correlation of voltage, current, and active power. As shown in Fig. 22 below, the selected node is the Xiangshawan 500 kV substation bus. There are a total of 9 branches connected to the Xiangshawan substation, which are labeled A-I. We will use a one-dimensional neural network to classify these 9 branches into three categories. Once a certain line fails or power fluctuates, we can predict the affected lines and identify associated weaknesses.

**Figure 23:**
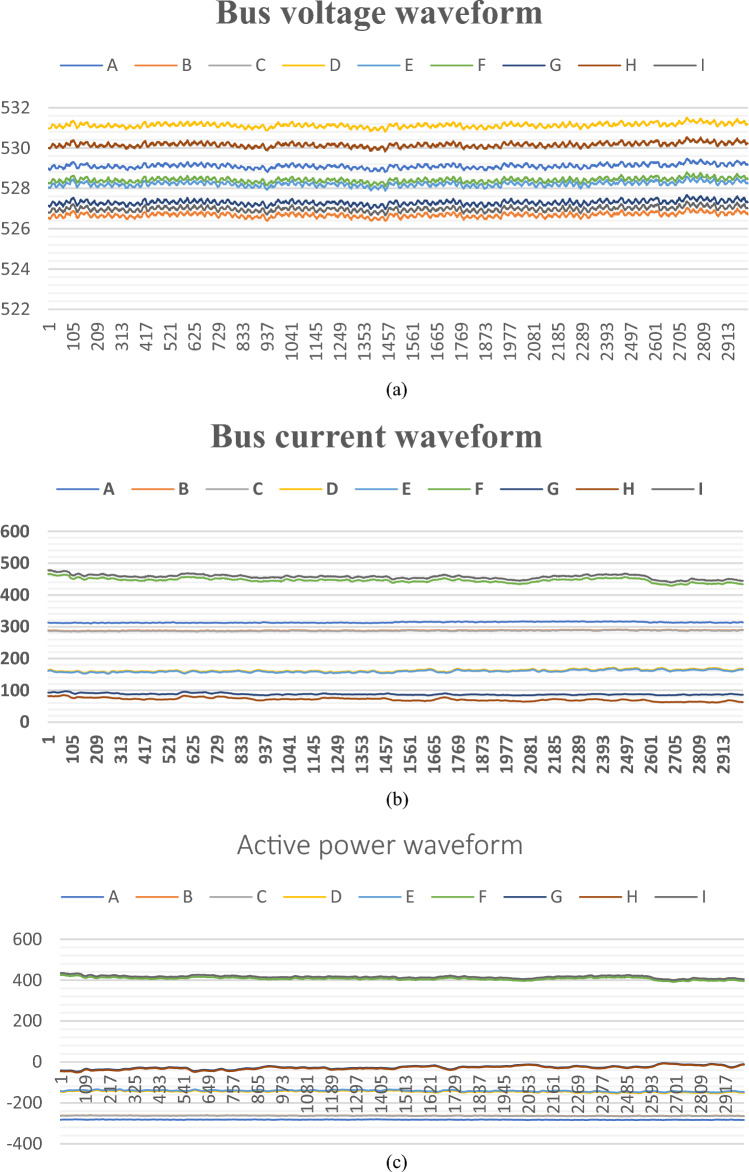
(a) Voltage Data of 9 Associated Lines at XiangshaWan Node. (b) Current Data of 9 Associated Lines at XiangshaWan Node. (c) Power data of 9 associated lines at XiangshaWan node.

As shown in Fig. 23 below, the same time section data recording and broadcasting for each branch is shown. Figure 23a shows the bus voltage, Fig. 23b shows the line current, and Fig. 23c shows the active power. We define the inflow as the negative direction, and the outflow as the positive direction. The training uses the first 2000 points of training data, and the last 1000 points of verification data. The simulation software uses MATLAB, and the hardware configuration is CPU: Core i9-12900 K, GPU: NVIDIA GeForce RTX 3090.

The key codes used for training are as appendix A. In this training, the number of hidden layer neurons is set to 10 layers, and the training set is further divided into training sets, verification sets, and test sets in a ratio of 70:15:15. According to the training, the correlation of the nine branches is shown in Table 2 below.

**Table 2:**
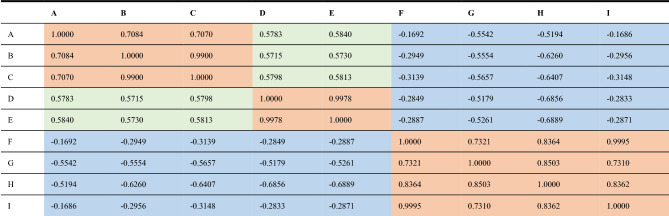
Correlation statistics of electrical quantities of Xiangshawan node connected to transmission lines.

**Figure 24:**
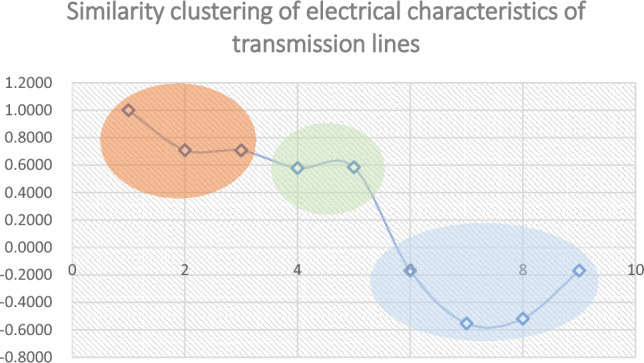
Transmission Line Correlation Classification.

Therefore, we can obtain the classification relationship of lines as shown in Fig. 24:

In the actual power grid, the three branches A, B, and C each come from three power plants, with the purpose of transmitting electrical energy through the Xiangshawan substation. Although the two lines D and E participate in the transmission of electrical energy, their fluctuations are affected by the load within the power supply section. The remaining line electrical parameter characteristics present a negative correlation and belong to the receiving end line. Therefore, this classification result is consistent with the actual situation. If disturbances or voltage stability anomalies occur, three groups of lines are classified into (A, B, C), (D, E), and (F, G, H, I), which exhibit electrical correlation.

## Discussion and conclusions

**Figure 25:**
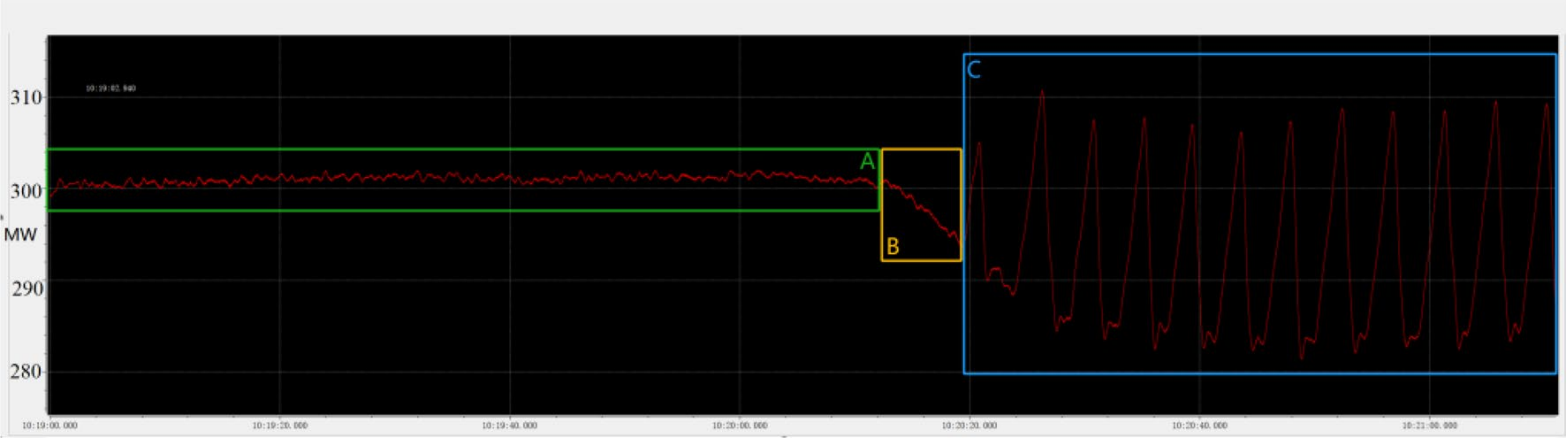
Wangqu Electric Field Unit 2 oscillation chart.

As shown in Fig. 25, The oscillation case data of Wangqu Electric Field Unit 2 on August 25, 2023 was from 10:19 to 10:21. In the C stage, the peak-valley difference of the oscillation was 24.714 MW, and the frequency of the oscillation was concentrated at 0.23 Hz.

- *Stage A* 10:19:00 ~ 10:20:12, this stage is defined as the pre-oscillation stable stage;
- *Stage B* 10:20:13 ~ 10:20:19, this stage is defined as the pre-oscillation mutation stage;
- *Stage C* 10:20:20 ~ 10:21:11, the second stage is defined as the oscillation stage

**Figure 26:**
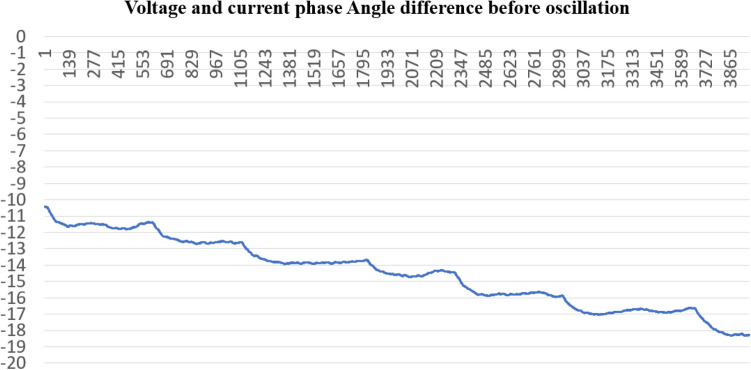
Phase angle characteristic curve of A stage before oscillation.

According to formula 19, the voltage and current phase Angle difference curve in Fig. 26 is obtained. We can conclude that the phase Angle difference of Unit 2 is continuously changing in the A stage before oscillation, ranging from − 10.4° to − 17.8°

**Figure 27:**
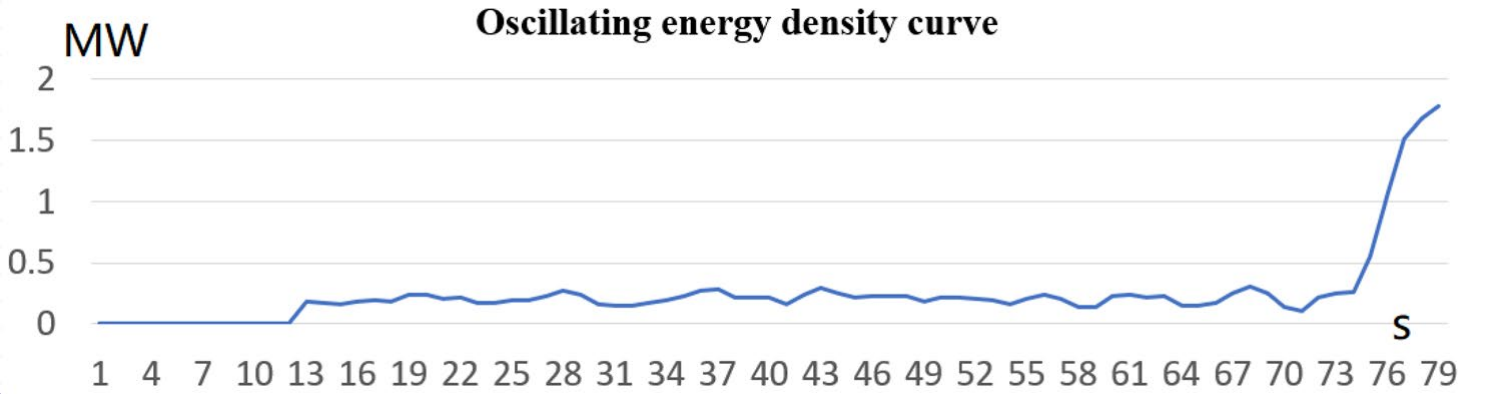
Oscillating energy density curve.

According to Formula 16, the disturbance energy density curve can be calculated as shown in Fig. 27. The amplitude of the disturbance energy density curve in stage A is very small, and the curve has A sudden change in stage B. Combined with Fig. 26, early warning can be made in stage A and stage B before the oscillation stage C.

According to the trend analysis theory of formulas 16–18, the risk warning module will give an early warning at about 10:20:10 s, which is about 52 s earlier than the oscillation time of 10:21:02.

To address the issue of broadband oscillations in ultra-high proportion new energy grid connected systems, it is necessary to predict, monitor, suppress oscillations, and develop corresponding equipment to eliminate system oscillations, thereby improving system stability and reliability. The research content of the article breaks through the current situation of oscillation suppression hysteresis, starting from the represented disturbance energy, gradually excavates the symptomatic characteristics of electrical quantities, and constructs physical and temporal correlations between electrical quantities.

The paper first explores the harmonic disturbance properties of power electronic equipment, and constructs typical oscillation scenarios through model simulation. In Fig. 10, we find that non convergent oscillation is an important situation that risk assessment focuses on. As shown in Fig. 5, for the divergence of disturbance energy, we constructed a power density curve and clearly found that disturbance energy had dense characteristics before oscillation occurred. In order to further lock in other electrical characteristics before oscillation, the article analyzed two factors: voltage current angle and impedance. At the same time, statistical analysis was conducted on the severe fluctuation of the active power curve, and the results showed that. In the case data, by analyzing impedance factors, the prediction lead of oscillation risk can be increased by 8 min. At the same time, we clearly see the time-domain performance of the three parameters during the oscillation development process, which is consistent with the risk assessment concept constructed in this article.

Subsequently, the paper conducted actual data analysis. By analyzing the power cross-section of the North China Power Grid in China, we analyzed the feature similarity of 9 lines. For the U/P/Q data of the power system, a one-dimensional convolutional neural network model structure with integrated attention mechanism was adopted to construct an implicit relationship mapping between voltage, active power, and reactive power at any time. Realized risk prediction and vulnerability analysis of new energy grid connected oscillation. The distribution of vulnerabilities can form a risk contour throughout the entire power supply network, achieving global risk assessment. In future research, we will continue to explore more sensitive and stable characterization methods based on the characteristics of electrical quantity data at different voltage levels and measurement points.

### Supplementary Information

Supplementary Information 1.

Supplementary Information 2.

Supplementary Information 3.

Supplementary Information 4.

Supplementary Information 5.

## Data Availability

The datasets generated and/or analysed during the current study are available in the [GitHub] repository, [https://github.com/users/titan27149/projects/1/views/1]. All data generated or analysed during this study are included in this published article [and its supplementary information files]. The file list is as follows: The very valuable real power system oscillation data in Guyuan area of China is used in the analysis of Figs. 5 and 13.csv. Figure 21a Voltage Data of 9 Associated Lines at Xiangsha Bay Node.csv. Figure 21b Current Data of 9 Associated Lines at Xiangsha Bay Node.csv. Figure 21c Power data of 9 associated lines at Xiangsha Bay node.csv.

## Abbreviations

CNN: Convolutional neural network
SSR/SSO: Sub-synchronous resonance/oscillation
DC: Direct current
DFIG: Double fed induction generator
RLC: Resistance inductance capacitance
GCN: Graph convolutional network
LSTM: Long short-term memory
DBN: Data base network
SDAE: Stacked denoising auto encoder
AC: Alternating current
HVDC: High voltage direct current
$C_{j}$: DC capacitance value of group j power electronic equipment
$V_{dcj}$: Voltage value of group j power electronic equipment
$I_{dj}$: Input current of the capacitor at the DC side of VSC-j
$I_{dcj}$: Output current of the capacitor at the DC side of VSC-j
$P_{j}$: Active power output of VSC-j
$Q_{j}$: Reactive power output of VSC-j
$V_{j}$: AC voltage of the AC side of VSC-j
$X_{fj}$: Filter reactance of the AC side of VSC-j
$I_{dj}$: D current of the AC side of VSC-j
$I_{qj}$: Q current of the AC side of VSC-j
$\vartriangle X_{acj}$: Vector of all the state variables of AC system j
$g_{pj} (s)$: Active power transfer function
$g_{qj} (s)$: Reactive power transfer function
$I_{{MTDC_{j} }}$: J-channel DC system power flow
$\Delta V_{dvi}$: Mapping model between new energy DC voltage and output active power
$$\rho$$: Power density
$\phi_{u - i}^{n}$: Voltage and current phase angle difference
G_g_(s): Transfer function equation of power electronic power supply station
G_s_(s): Transfer function equation of AC power grid
$O_{i}$: Obtained attention score
$x_{m}^{n}$: Represents the m-th input of layer n in the network,
$s_{i}^{n - 1}$: Represents the i-th output of layer n-1,
$w_{im}^{n - 1}$: Represents the convolution kernel of the corresponding channel $s_{i}^{n - 1}$
$N_{n - 1}$: Is the number of outputs $s_{i}^{n - 1}$ of layer n-1
$\otimes$: Represents the convolution operation
$\sigma (x_{m}^{n} )$: Represents the activation function,
$y_{m}^{n}$: Represents the output result
